# Supplementary figures and images for: Dynamic modelling of cell cycle arrest through integrated single-cell and mathematical modelling approaches
Source: PLoS Comput Biol. 2025 Oct 7;21(10):e1012890. doi: 10.1371/journal.pcbi.1012890 (PMC12520361; doi:10.1371/journal.pcbi.1012890)

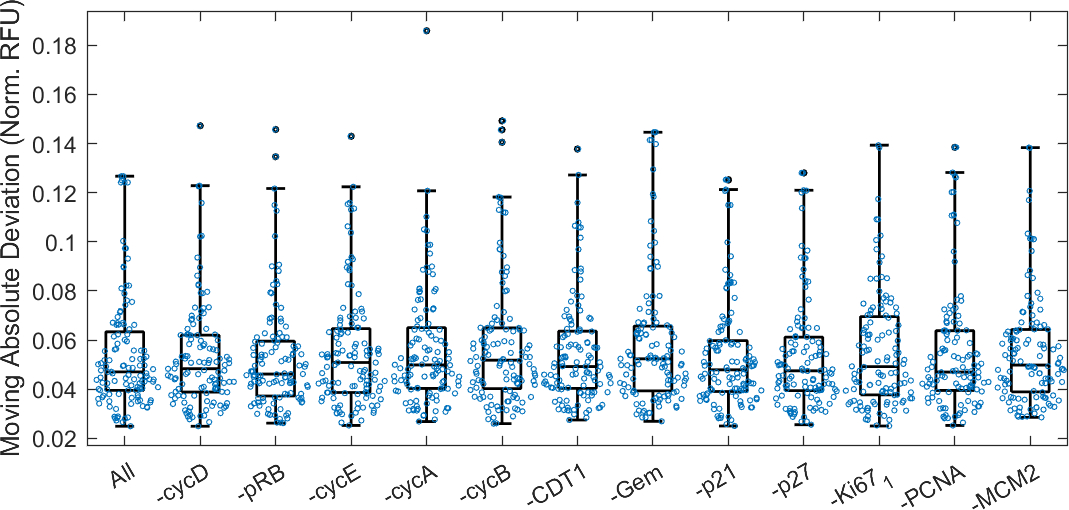

Supplement: S1 Fig — (TIF) [file pcbi.1012890.s001.tif]

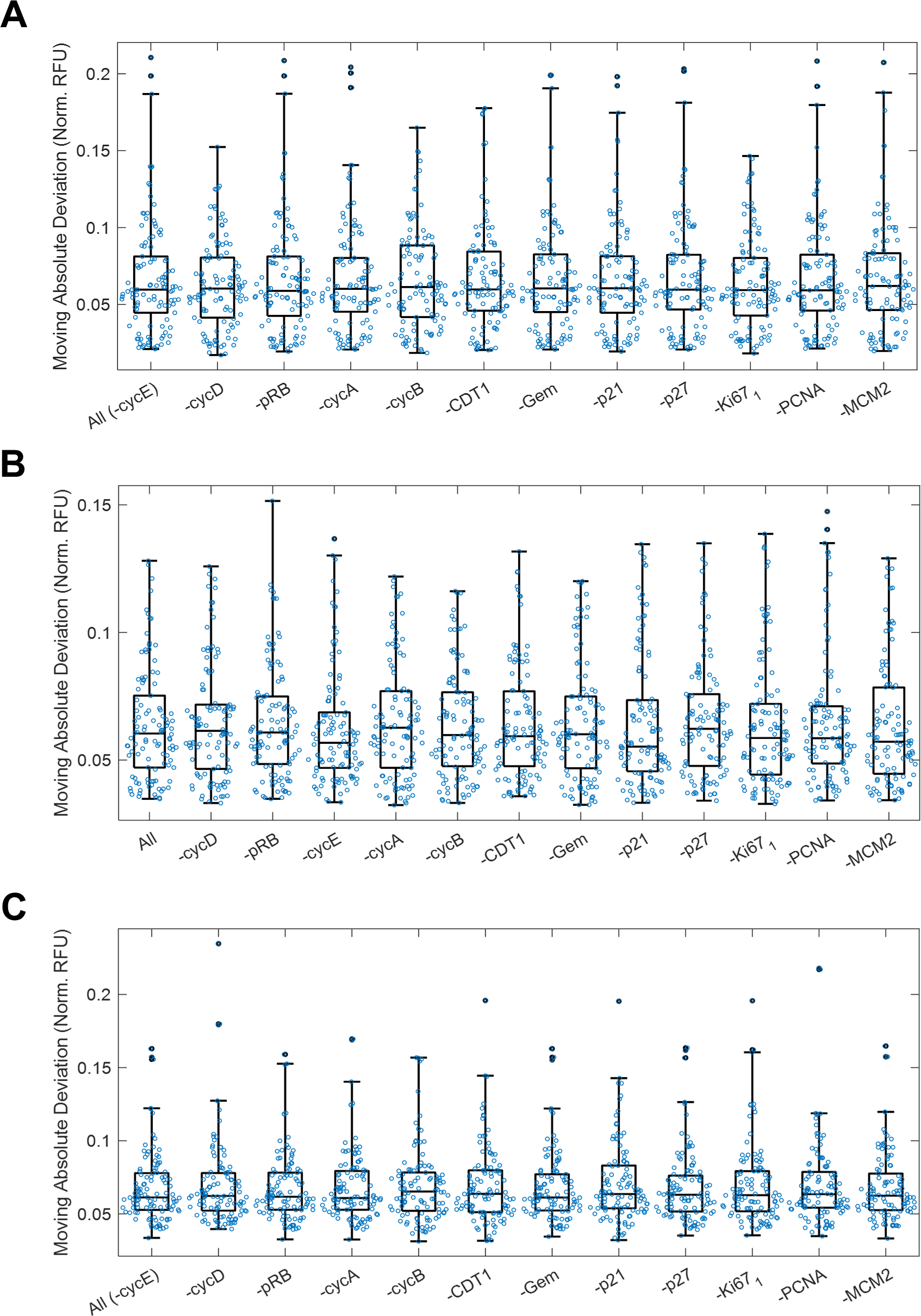

Supplement: S2 Fig — (A) ER+ cells. (B) TNBC cells. (C) HER2 + cells. (TIF) [file pcbi.1012890.s002.tif]

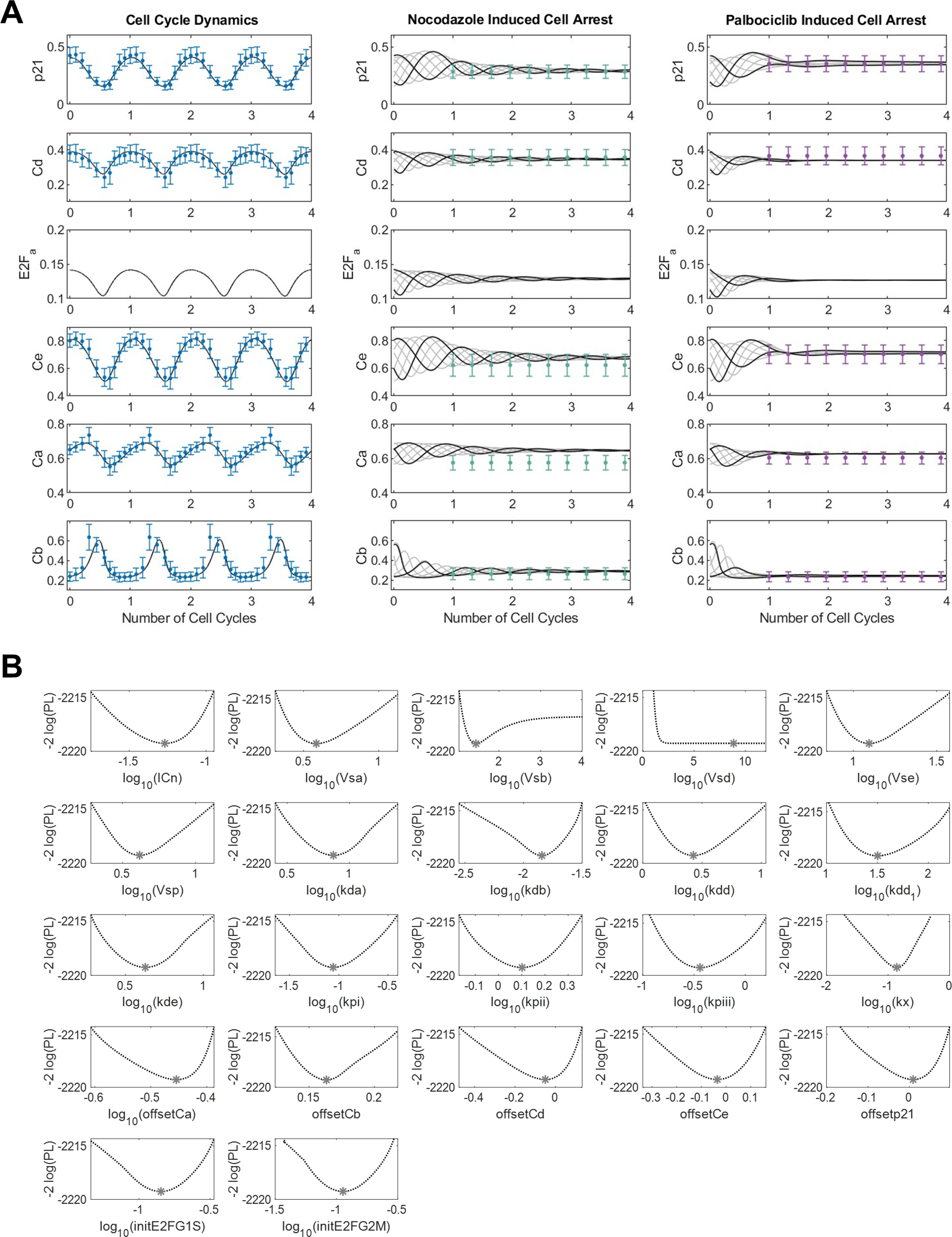

Supplement: S3 Fig — (A) Model fitting to cell cycle marker dynamics obtained from untreated and treated MCF-10A cells processed data using a non-ergodic approach to infer the dynamics. (B) Profile likelihood (PL) for each estimated parameter (black dotted line) and estimated parameter value (gray asterisk). (TIF) [file pcbi.1012890.s003.tif]

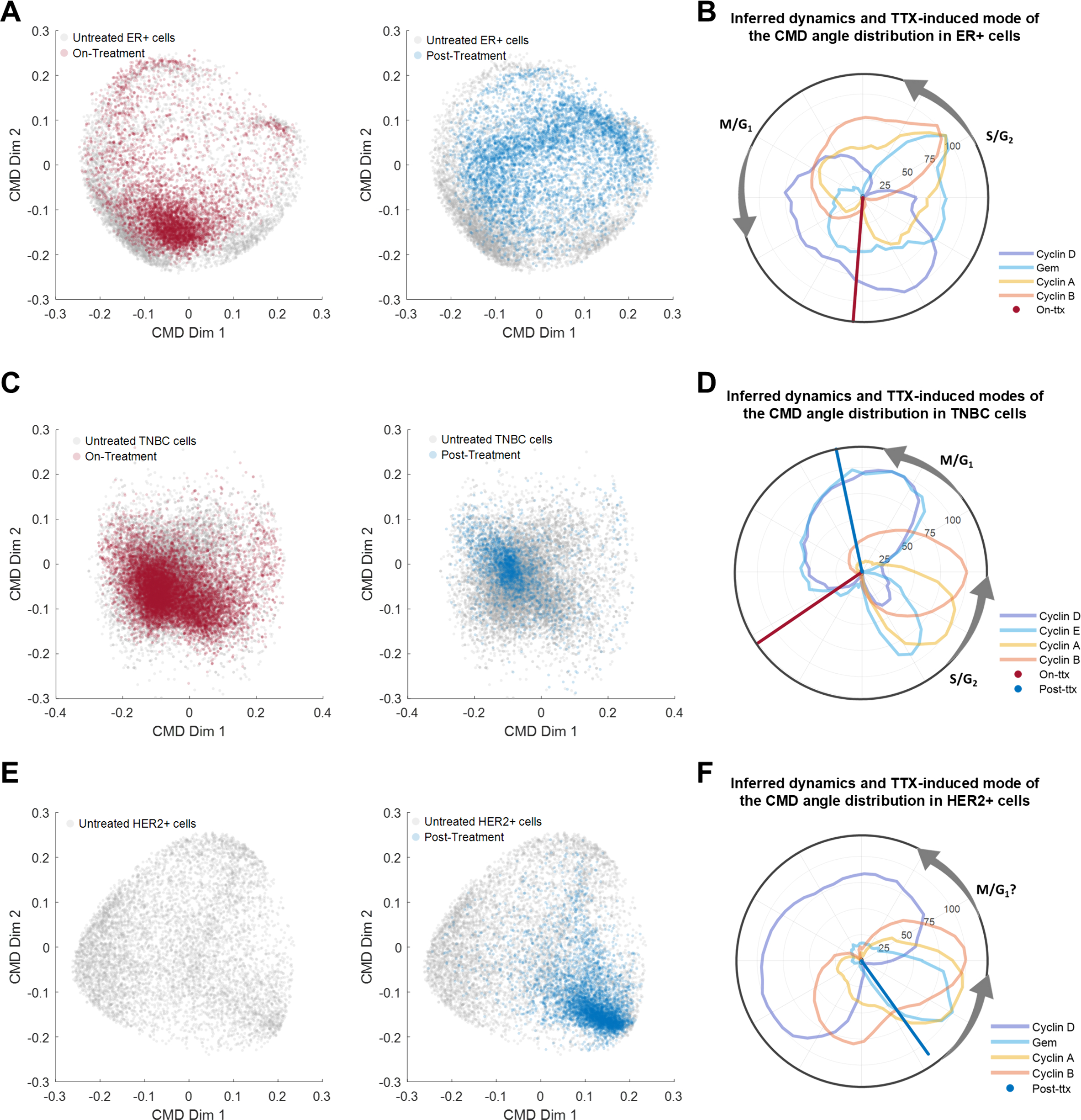

Supplement: S4 Fig — (A) CMD results for ER+ cells pre- (grey) on- (red) and post-treatment (blue). (B) Polar plot of normalized Cyclin D, A, B and Geminin dynamics in untreated ER+ cells, with angles mode during tamoxifen/aromatase inhibitor treatment (red line). (C) CMD results for TNBC cells pre- (grey) on- (red) and post-treatment (blue). (D) Polar plot of normalized Cyclin D, E, A and B dynamics in untreated TNBC cells, with angles modes of cells on-treatment with paclitaxel (red) and post-treatment with doxorubicin and cyclophosphamide (blue). (E) CMD results for HER2 + cells pre- (grey) and post-treatment (blue). (F) Polar plot of normalized Cyclin D, A, B and Geminin dynamics in untreated HER2 + cells, with angles mode post-treatment with pertuzumab and ado-trastuzumab emtansine (blue line). (TIF) [file pcbi.1012890.s004.tif]

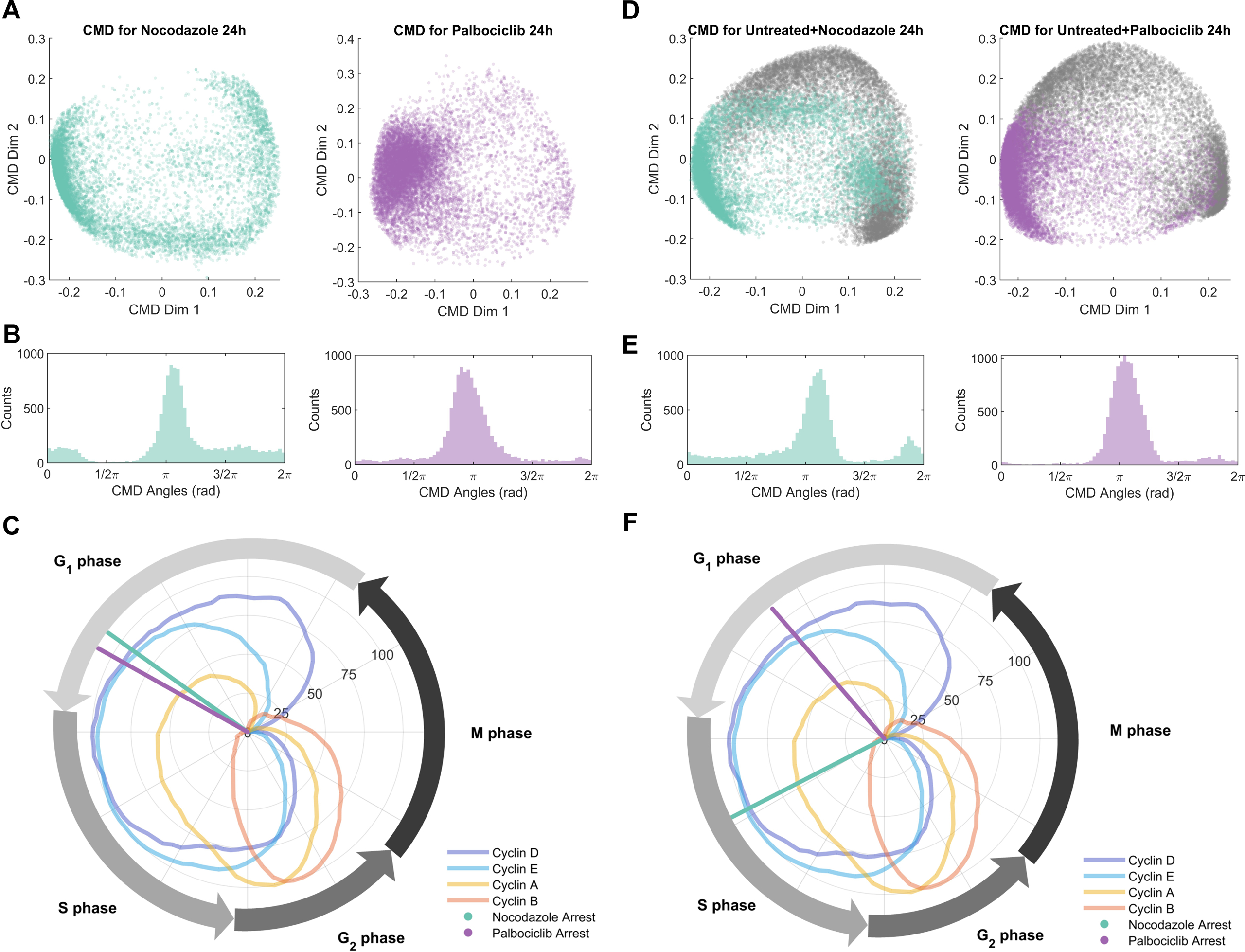

Supplement: S5 Fig — (A, D) CMD transformation results for MCF-10A cells treated with nocodazole on the left and palbociclib on the right. Every dot corresponds to a single cell, in green nocodazole treated cells, in purple palbociclib treated cells and in grey untreated cells. (B, E) Histogram of CMD angles distribution for nocodazole on the left and palbociclib on the right show unimodal distributions that were used to map the corresponding cell cycle arrest times. (C, F) Polar coordinate plot of normalized cyclin dynamics (untreated cells) and cell arrest times for nocodazole and palbociclib. Radial coordinate corresponds to cell cycle time from untreated cell dynamics. Polar coordinate corresponds to moving median of marker levels min-max normalized to the interval [0,100]. Cell cycle phases between checkpoints were manually annotated based on cyclin dynamics obtained previously. (TIF) [file pcbi.1012890.s005.tif]

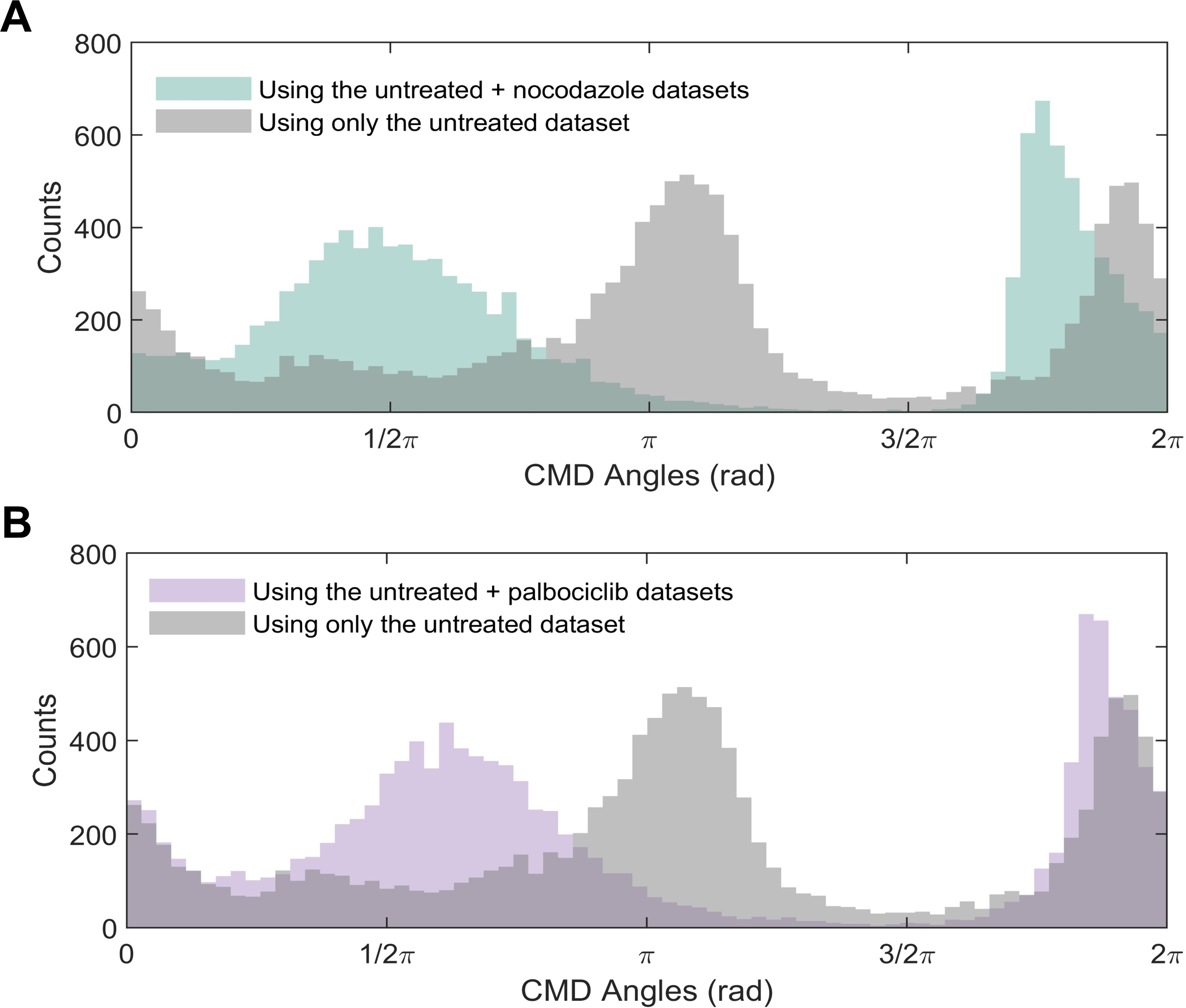

Supplement: S6 Fig — Angle distribution results using data processing results from CMD performed with untreated data only (gray) versus untreated + treated datasets used at once. Light green for untreated + nocodazole datasets (A), and light purple for untreated + palbociclib datasets (B). (TIF) [file pcbi.1012890.s006.tif]

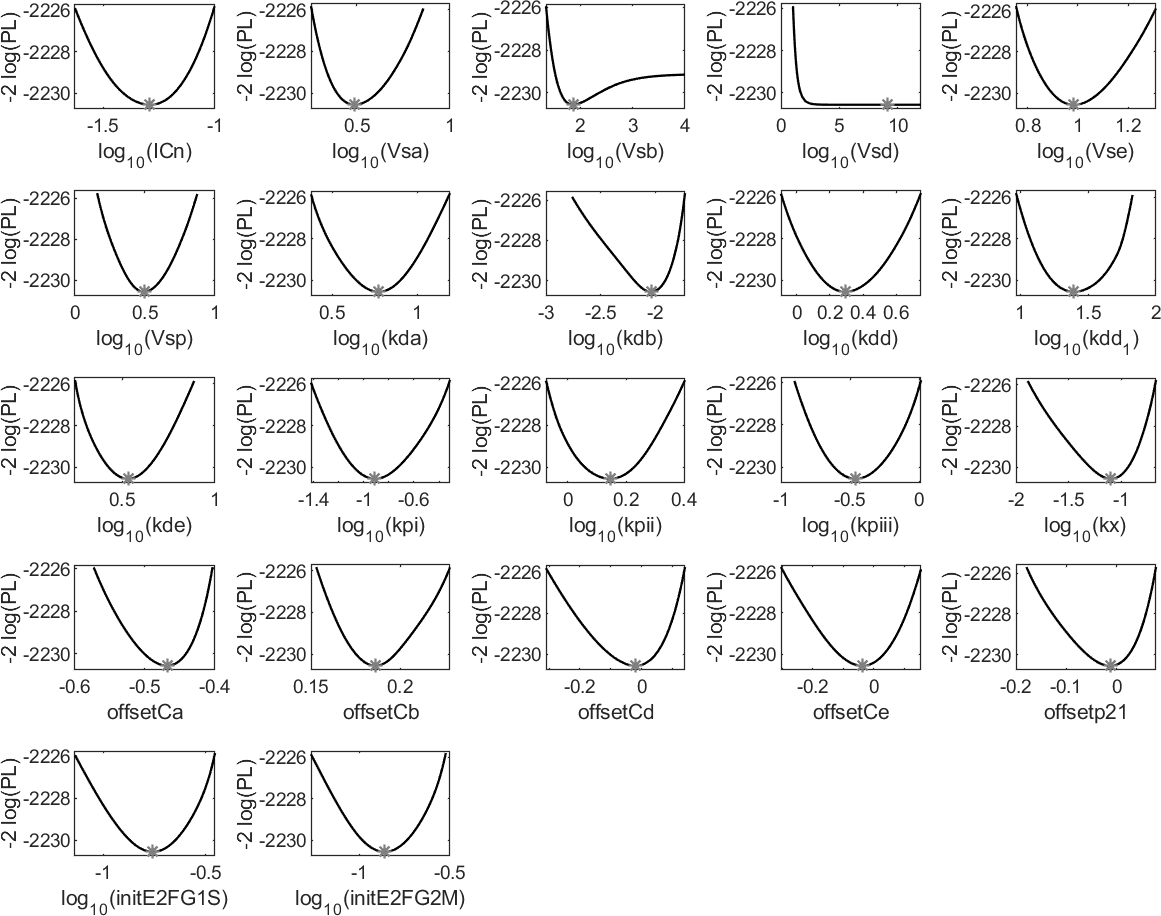

Supplement: S7 Fig — Profile likelihood (PL) for each estimated parameter (black line) and estimated parameter value (gray asterisk). (TIF) [file pcbi.1012890.s007.tif]

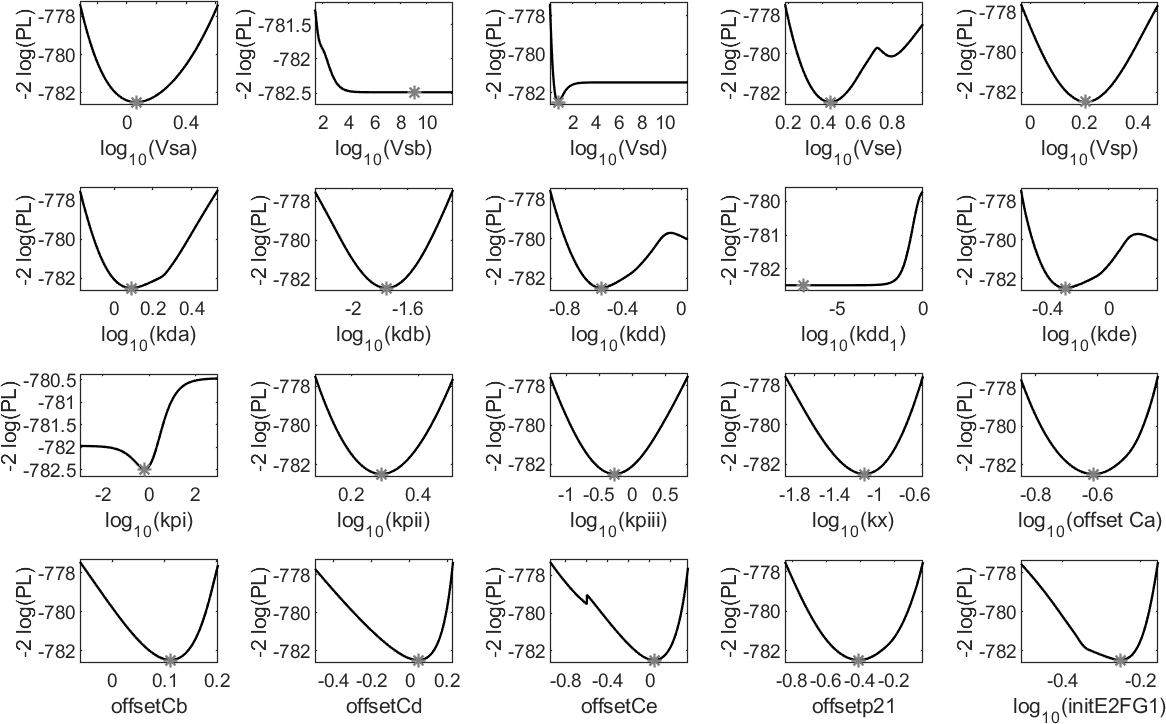

Supplement: S8 Fig — Profile likelihood (PL) for each estimated parameter (black line) and estimated parameter value (gray asterisk). (TIF) [file pcbi.1012890.s008.tif]

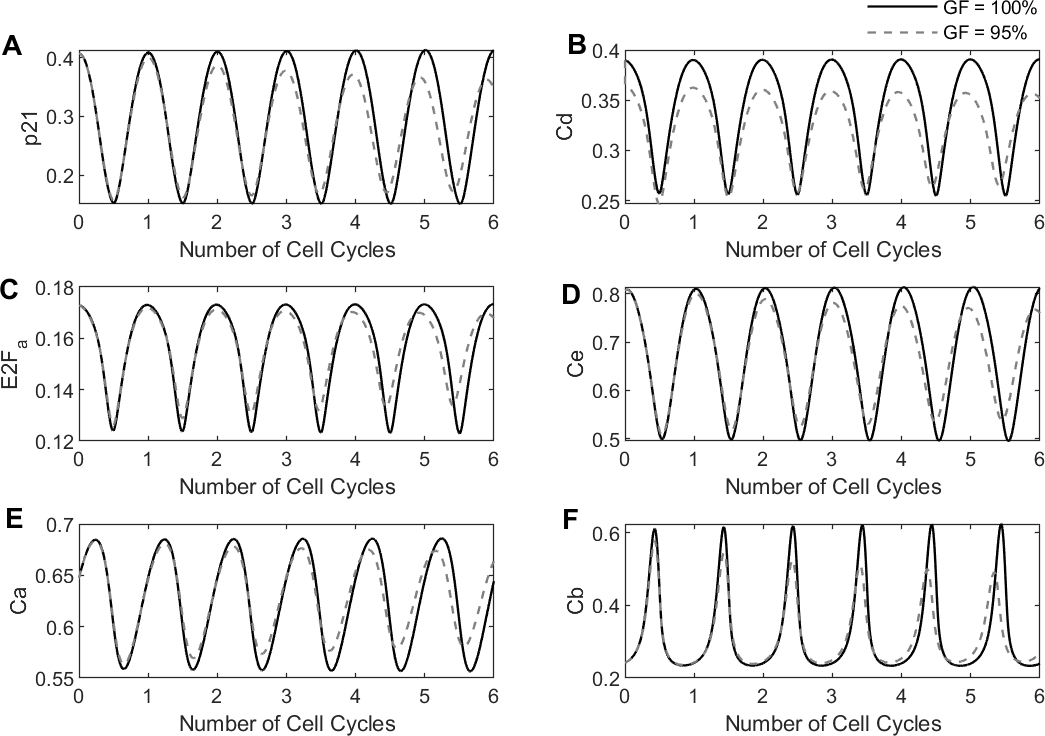

Supplement: S9 Fig — Predicted dynamics across six cell cycles of the state variables (A) p21, (B) Cd, (C) E2Fa, (D), Ce (E) Ca and (F) Cb sing GF = 100% (black lines) and GF = 95% (dashed gray lines). (TIF) [file pcbi.1012890.s009.tif]

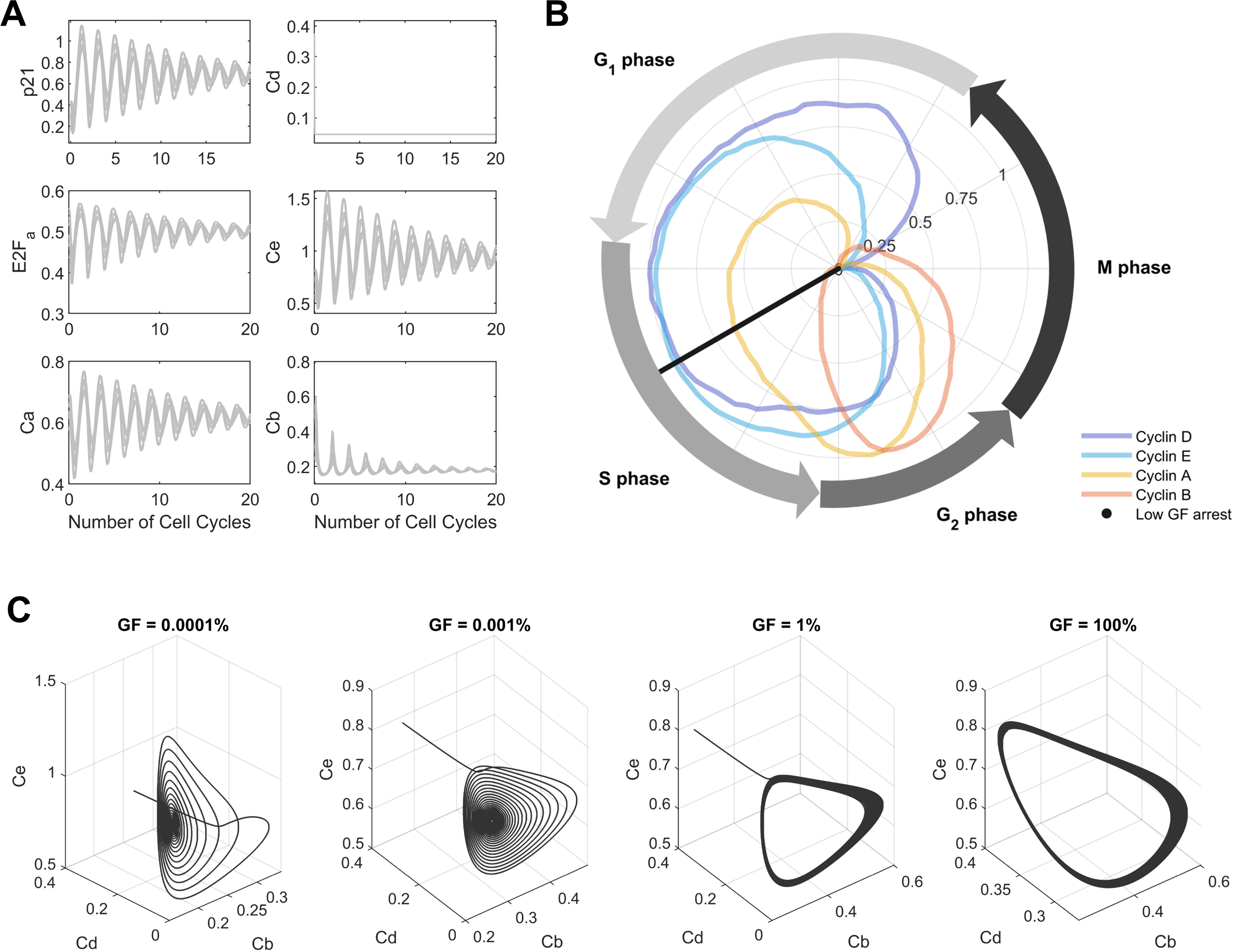

Supplement: S10 Fig — (A) Predicted dynamic of the state variables p21, Cd, E2Fa, Ce, Ca and Cb for different starting points of the cell cycle (initial conditions) lead to the same cell arrest state for GF = 0.0001%. (B) Polar coordinate plot of normalized cyclin dynamics (untreated cells) and arrest time predicted for GF = 0.0001% at S phase. Polar coordinate corresponds to moving median of marker levels min-max normalized to the interval [0,100]. Cell cycle phases between checkpoints were manually annotated based on cyclin dynamics obtained previously. (C) Simulations for Ce, Cd and Cb variables at different growth factor (GF) levels (0.0001, 0.001, 1 and 100%) reveal a limit cycle for high GF values (≳ 1%) and a cell arrest at lower GF values. (TIF) [file pcbi.1012890.s010.tif]
