# Supplementary material for: Dynamic modelling of cell cycle arrest through integrated single-cell and mathematical modelling approaches
Source: PLoS Comput Biol. 2025 Oct 7;21(10):e1012890. doi: 10.1371/journal.pcbi.1012890 (PMC12520361; doi:10.1371/journal.pcbi.1012890)
Supplement: S1 Text — (DOCX) [file pcbi.1012890.s012.docx]

**Processing of multiplexed immunofluorescence data from biopsy samples**

We applied the CMD ordering method to published biopsy data [1], which posed greater challenges than cell line data due to higher noise levels, smaller sample sizes, and differing growth conditions. In the case of ER+ cells, despite the absence of cyclin E measurements, the method applied using the cell cycle marker vector excluding only cyclin E successfully reconstructed the marker dynamics (**S4A-B Fig**). However, for triple-negative breast cancer (TNBC) and HER2+ cell samples, the method was less effective, failing to produce coherent trajectories spanning the entire cell cycle (**S6C-F Fig**). Given the lack of reference dynamics for these specific cell types, it is difficult to determine whether the observed variability reflects true biological differences or results from limitations of the CMD ordering method. Nevertheless, we provide a comparison with classical cell cycle dynamics reported in the literature [2,3]. For TNBC cells, the cyclin trajectories displayed a pattern roughly consistent with expectations; cyclins D and E increased first, followed by peaks in cyclins A and B. However, unlike the typical sequence where cyclin D rises before cyclin E, both increased simultaneously in this case, with cyclin E showing a biphasic pattern, rising again just before the cyclin A peak. In the case of HER2+ cells (lacking cyclin E data), the trajectories showed an initial increase in cyclin D, followed by an early peak in cyclin B, and subsequent peaks in geminin, cyclin A, and a second cyclin B peak, resulting in an almost symmetrical ordering. It is possible that cells with similar levels of cyclins D and B were ordered interchangeably at these positions, suggesting that the inclusion of cyclin E or another discriminating marker could enhance the resolution and accuracy of the proposed method in this case. Subsequently, we applied the CMD embedding method to treatment conditions, revealing G1-phase arrest in on-treatment ER+ and TNBC cells, no arrest in ER+ post-treatment cells (as indicated by the absence of a distinct peak in the angle distribution), G1-phase arrest in TNBC post-treatment cells, and a potential S/G2 or G2/M arrest in HER2+ cells. Overall, these findings highlight both the potential and the current limitations of the CMD approach when applied to more heterogeneous and complex clinical data.

**References**

1. Gaglia G, Kabraji S, Rammos D, Dai Y, Verma A, Wang S, et al. Temporal and spatial topography of cell proliferation in cancer. Nature Cell Biology 2022 24:3. 2022;24: 316–326. doi:10.1038/s41556-022-00860-9
2. Hochegger H, Takeda S, Hunt T. Cyclin-dependent kinases and cell-cycle transitions: does one fit all? Nature Reviews Molecular Cell Biology 2008 9:11. 2008;9: 910–916. doi:10.1038/nrm2510
3. Alberts B, Heald R, Johnson A, Morgan D, Raff M, Roberts K, et al. Molecular Biology of the Cell. Senventh. W. W. Norton & Company; 2022.
