# Supplementary material for: Dynamic modelling of cell cycle arrest through integrated single-cell and mathematical modelling approaches
Source: PLoS Comput Biol. 2025 Oct 7;21(10):e1012890. doi: 10.1371/journal.pcbi.1012890 (PMC12520361; doi:10.1371/journal.pcbi.1012890)
Supplement: Attachment — Submitted filename: Response to Reviewers.pdf [file pcbi.1012890.s014.pdf]

Below, we provide a detailed point-by-point response that addresses all of the concerns raised by the reviewers. Reviewers comments is provided in boxes with yellow shading, our responses are in boxes with white shading and changes to the text are presented in boxes with red shading.

Reviewer #1

Reviewer 1:
The paper by Cortés-Ríos et al presents an integrated framework combining single-cell multiplexed immunofluorescence data with mathematical modelling to decipher cell cycle dynamics under both oscillatory and arrest conditions. The authors extend the cc-CMD pseudo-time ordering method to integrate data from multiple experimental conditions, including treatments with palbociclib and nocodazole. By incorporating regulatory interactions such as p21/p27 inhibition of CDK-cyclins and the impact of cyclin B on p21 degradation, the model successfully recapitulates the cell cycle progression of untreated MCF 10A cells and accurately predicts arrest states corresponding to early G1. Model calibration using both untreated and drug-treated datasets reduced parameter uncertainty and yielded biologically plausible simulations. The predictive capacity of the model was further validated under growth factor starvation, demonstrating its potential for extrapolating cell cycle arrest conditions not included in the training set. The paper presently a sensible approach though I am not 100% sure of some of the assumptions made and the extensibility of the approach to other datasets.

Response: We thank the reviewer for their insightful comments. We believe that the revisions and additional results now included help clarify the underlying assumptions and demonstrate the extensibility of the proposed framework.

Major points

Reviewer 1 (Major Point 1): The method is inherently based on the assumption of ergodicity, i.e. that the cell population is representative across all phases in proportion to the time spent in each phase—which may not hold true for unsynchronized cells or those grown under non-ideal conditions. If this assumption fails, the inferred cell cycle times and phase transitions could be significantly skewed. Can the authors test the effect on their method's output by using simulated data instead of experimental data, e.g. using the stochastic simulation algorithm, that is not in quasi-steady state conditions and that hence does not obey the ergodic hypothesis.

Response: We acknowledge and thank the reviewer for raising this point. Rather than using stochastic simulations, we incorporated model fitting results based on an alternative time vector inference approach. Specifically, we used the equation published by Kafri et al. (2013) to estimate the cell cycle time, which does not assume ergodicity and is specifically designed for expanding cell populations. The method infers time from cell count data using the formula:

$$t = \frac{T}{\log(2)} \log\left(\frac{2N}{2N - r}\right)$$

In this equation, T represents the doubling time, N is the total number of cells, and r is the number of cells younger than time t. The model fitting results and corresponding profile likelihoods are presented in S3 Figure. Using this approach, the inferred marker dynamics are accelerated for early cell cycle phases and decelerated for late cell cycle phases compared to those obtained with the previous method. However, the model achieves a similar quality of fit, with nearly identical profile likelihoods across all estimated parameters and comparable parameter values (see S1 Table). These results support the applicability of the method under different time inference methods.

Original text: This approach assumes that the sample of cells analysed contains cells in different cell cycle phases, with population densities proportional to the time spent in each phase (mean sojourn time), a common assumption in single-cell data analysis (this is effectively an assumption that the data are ergodic [45]). We note that this assumption has been challenged in the context of cells not grown to full confluency [23,27], but alternative theoretical frameworks also fail to account for cell death.

Revised text: This approach assumes that the sample of cells analysed contains cells in different cell cycle phases, with population densities proportional to the time spent in each phase (mean sojourn time), a common assumption in

single-cell data analysis (this is effectively an assumption that the data are ergodic [45]). We note that this assumption has been challenged in the context of cells not grown to full confluency [23,27], but found that time inference methods that do not rely on ergodicity yield comparable reconstructed marker trajectories, with negligible influence on downstream analyses (**S3 Figure**, **S1 Table**).

New text (Methods section): To enable comparison between different time inference approaches, we employed the equation proposed by Kafri et al. [71] (equation 1) to estimate the time vector (t) associated with the marker trajectories:

$$t = \frac{T}{\log(2)} \log\left(\frac{2N}{2N - r}\right) \qquad Eq. 1$$

Where T is the doubling time, N is the total number of cells and r is the number of cells younger than t. For this approach, we used the moving median to the inferred time vector that corresponded to the interpolations of the moving average of marker concentrations of cell cycle markers.
Additionally, CMD scaling method was applied to biopsy data from ER+ (Case 08 and Case 06, N = 5605 untreated/treated cells), HER2+ (N = 6620 untreated and 4695 post-treated cells) and TNBC cells (12000 sampled cells for pre- and on-treatment phases and 2250 cells for the post-treatment period). CMD embedding was performed for each data set for on- and post-treatment data when available. The mode of the angular distribution on- and/or post-treatment was obtained and plotted alongside cell cycle marker dynamics using the original ergodic approach (Cyclins D, A, B, and E when available, or Geminin when Cyclin E was unavailable).

Reviewer 1 (Major Point 2): Another potential issue is the reliance on a single MCF-10A cell line and a restricted marker panel, which raises concerns about the generalizability of the findings to other cell types or biological contexts. Would be useful to apply to data beyond this particular cell line to remove these concerns.

Response: We agree that the use of only one cell line (MCF-10A) is a limitation of the study. Unfortunately, we do not have access to comparable datasets from other cell lines to perform a broader analysis. Instead, we incorporated results using available biopsy data from ER+, HER2+, and triple-negative breast cancer (TNBC) samples (S4 Figure). However, these datasets are inherently more complex than cell line data due to higher noise levels, smaller sample sizes, and differences in growth conditions. These factors introduce significant challenges for applying the method. While the approach successfully ordered ER+ cells and identified G1 phase arrest during treatment (S4A and S4B Figures), it was less effective for reconstructing TNBC and HER2+ dynamics. We have acknowledged these limitations in the Discussion section (lines 340 – 345).

Original text (Results): Subsequently, cell cycle time inference suggested that cells spend approximately two thirds of the cell cycle time in G1+S phases, and one third in G2+M phases, which is consistent with prior reports with for MCF-10A cells [47,48]. Overall, this confirms that CMD-based pseudo-time ordering of cell-cycle data is robust and generates biologically plausible trajectories.

Revised text (Results): Subsequently, cell cycle time inference suggested that cells spend approximately two thirds of the cell cycle time in G1+S phases, and one third in G2+M phases, which is consistent with prior reports with for MCF-10A cells [47,48]. In contrast, trajectory construction for patient-derived data, characterized by higher noise, fewer single cells (<7,000 in ER+ and HER2+ samples) and lower coverage of cell-cycle markers (missing cyclin E in ER+ and HER2+ samples), was less reliable and showed poorer agreement with previous reports (see Supplementary Information Section “Supplementary Results”, **S4 Figure**), and was therefore excluded from further analysis in this study. Overall, this confirms that, given sufficient data quality and quantity, CMD-based pseudo-time ordering of cell-cycle data is robust and generates biologically plausible trajectories.

New text (Discussion): While the proposed framework proved robust when applied to MCF-10A data, we observed some limitations in capturing cyclin A and B dynamics with high precision. Specifically, model simulations for these markers showed minor deviations from the experimental data for a few data points, likely due to either unmodeled regulatory mechanisms or the limitations of the CMD-based trajectory reconstruction itself. This limitation becomes more pronounced in the case of biopsy data, where the CMD ordering method, though effective in providing ordered trajectories in ER+ cells, remains an approximation and cannot be considered a fully precise representation of true cell cycle progression. The increased complexity, noise, and heterogeneity in biopsy samples (compared to cell line data) can compromise the accuracy of pseudo-time ordering and downstream modeling. These factors should be considered when interpreting model outputs from such datasets.

Reviewer 1 (Major Point 3): The method appears to completely ignore the inherent large cell-to-cell variability in single cell data (as they use the moving median for the protein levels). While noise can be seen as a nuisance, actually there are many studies (especially in the scRNA sequencing literature) which point out that it possesses biological information that is better used rather than discarded. The authors should discuss how they would include this variability in the protein levels and the anticipated effect on their method and on the main results reported in this paper. In this context, the literature on the use of stochastic models of the cell-cycle (especially those used in conjuction with single-cell data) would probably be useful to consult and to reference appropriately.

Response: We thank the reviewer for this comment. We have included in the Discussion section (lines 311 - 316) that cell-to-cell variability or noise could be addressed in future work by applying nonlinear mixed-effects models, which incorporate both fixed and random effect parameters, or by using stochastic modeling approaches to explicitly account for this variability, and we have added the corresponding references.

Original text: Additionally, quantifying responses to more perturbations across a larger set of cell lines would be essential for constructing more comprehensive models that are applicable to different cell types. To this end, the pseudo-time ordering approach we developed could easily be adapted to work with data from multiple cell-lines.

Revised text: Additionally, quantifying responses to more perturbations across a larger set of cell lines would be essential for constructing more comprehensive models that are applicable to different cell types. Moreover, cell-to-cell variability, which reflects biologically relevant differences in cell cycle dynamics and drug responses, could explicitly be addressed in future work by applying nonlinear mixed-effects models that incorporate both fixed and random effect parameters [61,62], or by using stochastic modeling approaches [63–66] that capture these fluctuations more directly. To this end, the pseudo-time ordering approach we developed could be adapted to stochastic modelling framework and work with data from multiple cell-lines.

Reviewer #2

Reviewer 2:
The title of the manuscript is misleading: it should highlight that the main contribution of this work is methodological, and not on modeling of cell cycle arrest. As far as I understand, the authors show that using pseudotime techniques on multiple experimental data via appropriate training provides accurate and relevant results, when applied to the modeling of cell cycle arrest. This is certainly an important result, that would deserve publication, provided that its relevance is better motivated.

Response: We thank the reviewer for their comments. We believe the main contribution of this manuscript is to provide a framework for integrating multiplexed immunofluorescence data with mathematical modeling across both perturbed and unperturbed conditions. The aim of this study is not to propose a new method for pseudo-time ordering, but rather to demonstrate how existing pseudo-time inference (with some modifications) can be integrated with a trainable and identifiable model. This integrated approach successfully predicts cell-cycle arrest in G1 under low growth factor conditions (not used in model training) and captures data in both cell growth and cell arrest states, which to the best of our knowledge, has not been demonstrated previously.

Major points

Reviewer 2 (Major Point 1): Lines 101-102, the authors claim to "develop novel model training techniques". Reading through the manuscript, it is unclear what is new, since the cc-CMD method is not new and already proved to be useful. Moreover, it is not clear what the extension of this method consists in. Since this is, in my opinion, the core of the current work, it should be clearer and discussed in more depth (advantages, limitations, etc.).

Response: We thank the reviewer for pointing us to an incomplete sentence with bad grammar, which evidently led to some confusion. The manuscript has two key methodological contributions:

(i) the extension of cc-CMD to multi-condition/perturbational data, which is required to incorporate data with drug treatments
(ii) the extension of training methods for mathematical models to periodic/oscillatory dynamics and steady-state data, which is required to describe untreated and treated data in a single model

We have rewritten the section referenced by the reviewer and further clarified our contributions throughout the manuscript.

Original text (Abstract): We devise training strategies that are applicable to datasets where cells exhibit oscillatory as well as arrested dynamics and use them to train a cell cycle model on a dataset of MCF-10A mammary epithelial exposed to cell-cycle arresting small molecules.

Revised text (Abstract): We devise training strategies for mathematical models that are applicable to datasets where cells exhibit oscillatory as well as arrested dynamics and use them to train a cell cycle model on a dataset of MCF-10A mammary epithelial exposed to cell-cycle arresting small molecules.

Original text (Introduction): In this work, we extend a cc-CMD pseudo-time ordering [9] to enable model training using multi-condition data, thereby enabling pseudo-time ordering of perturbational data. Additionally, we develop novel model training techniques that can accommodate both oscillatory and steady-state constraints. We apply this framework to train a mathematical model of the cell cycle using previously described single-cell multiplexed immunofluorescence data [9]. We apply this framework to train a mathematical model of the cell cycle using previously described single-cell multiplexed immunofluorescence data on cell cycle progression and drug-induced cell arrest by palbociclib and nocodazole in MCF-10A cells [9]. Finally, we demonstrate that the trained model accurately replicates oscillatory and arrested cell cycle dynamics in training data and predicts cell arrest states for cells at different starting points of the cell cycle as well as G1 arrest after growth factor depletion.

Revised text (Introduction): In this work, we extend a cc-CMD pseudo-time ordering approach [9] to multi-condition data, thereby enabling pseudo-time ordering of perturbational data. Additionally, we develop a novel training technique for mathematical models that can incorporate oscillatory and steady-state constraints. We apply the extended cc-CMD approach along with this training method for mathematical models to multiplexed single-cell immunofluorescence data from MCF-10A cells [9], enabling the training of a mechanistic model of the cell cycle under both untreated and drug-induced arrested conditions by palbociclib and nocodazole. We demonstrate that only the trained model on both conditions accurately replicates oscillatory and arrested cell cycle dynamics in training data and predicts cell arrest states for cells at different starting points of the cell cycle as well as G1 arrest after growth factor depletion.

Original text (Results): Data from both untreated and treated conditions were used for parameter estimation. To encourage the model to exhibit stable oscillations under treated conditions, we concatenated four copies of the dynamic trajectories of all markers. To encourage model simulation to converge to a steady state under treated conditions, we concatenated 10 temporally equally spaced copies of the moving average marker concentrations at the cell cycle time of the corresponding arrest between the end of the first and the fourth cell cycle.

Revised text (Results): Data from both untreated and treated conditions were used for parameter estimation. The proposed model training approach builds on maximum likelihood estimation, with specific conditions for fitting oscillatory systems under drug-induced arrest. Specifically, to encourage the model to exhibit stable oscillations under treated conditions, we concatenated four copies of the dynamic trajectories of all markers. To encourage model simulation to converge to a steady state under treated conditions, we concatenated 10 temporally equally spaced copies of the moving average marker concentrations at the cell cycle time of the corresponding arrest between the end of the first and the fourth cell cycle.

Reviewer 2 (Major Point 2): Lines 160-162, the authors claim their results "confirm" the relevance of CMD-based pseudo-time techniques, yet, as mentioned above, this was already known. Can the authors clarify what is confirmed by their results?

Response: While cc-CMD has previously been used for pseudotime analysis, the evaluation of biological plausibility was relatively limited. In the section "Processing of multiplexed immunofluorescence data allows reconstruction of the dynamics of the main cell cycle proteins", we present new analyses that include:

(i) an assessment of trajectory robustness through the removal of individual markers, and
(ii) a comparison of inferred cell cycle durations with values reported in the literature.

We do not consider these analyses to be central contributions of the paper, and we hope we have not overstated their importance. Nonetheless, they offer a gentle introduction for readers—particularly those within the broad PLOS Computational Biology audience who may be less familiar with cell cycle biology or pseudotime methods.
To further underscore the relevance of these analyses, we now include complementary results on patient data in the supplementary material (S1 and S2 Figures and Supplementary Results section). In that case, we found the reconstructed trajectories to be insufficiently reliable for proceeding with mechanistic modeling.

Original text: Statistical tests showed no significant differences (p-values > 0.05, using the Benjamini & Hochberg (1995) procedure for controlling the false discovery rate for multiple-testing [43], **S1 Figure**), indicating that the CMD embedding is robust to the removal of individual markers. We therefore used all 12 available cell-cycle markers to perform the CMD; these corresponded to cyclin D, cyclin E, cyclin A, cyclin B, pRB, CDT1, geminin (Gem), p21, p27, Ki67, PCNA, and MCM2.

Revised text: Statistical tests showed no significant differences (p-values > 0.05, using the Benjamini & Hochberg (1995) procedure for controlling the false discovery rate for multiple-testing [43], **S1 Figure**), indicating that the CMD embedding is robust to the removal of individual markers. The same analysis was performed on published biopsy datasets for ER+, triple-negative (TNBC), and HER2+ breast cancer samples [9] which also showed no significant changes upon removal of a single marker (**S2 Figure**). We therefore used all available cell-cycle markers to perform the CMD; these corresponded to cyclin D, cyclin E (excluded for ER+ and HER2+ samples due to missing data), cyclin A, cyclin B, pRB, CDT1, geminin (Gem), p21, p27, Ki67, PCNA, and MCM2.

Reviewer 2 (Major Point 3): Lines 231-232, the authors mention that "the simulations for cyclin B and cyclin A were slightly outside the median absolute deviation range for a few datapoints", could they justify this result or at least hypothesize why this is observed? It is probably the most interesting unexpected result of their work, I believe it deserves more attention.

Response: We thank the reviewer for this insightful comment. We acknowledge this as a limitation of the framework, which simplifies complex biological processes into a reduced model. Possible explanations include the presence of additional regulatory mechanisms not incorporated in the model that may influence cyclin dynamics in MCF10A cells, intrinsic biological variability, or measurement noise affecting the data. Additionally, the CMD ordering method and time inference enables reconstruction of ordered trajectories; however, it may still exhibit small deviations and should not be considered a fully precise representation of the underlying cell cycle dynamics. These factors collectively may contribute to the deviations observed between simulations and experimental data. We have discussed these limitations in the Discussion section, lines 336 – 340.

New text (Discussion): While the proposed framework proved robust when applied to MCF-10A data, we observed some limitations in capturing cyclin A and B dynamics with high precision. Specifically, model simulations for these markers showed minor deviations from the experimental data for a few data points, likely due to either unmodeled regulatory mechanisms or the limitations of the CMD-based trajectory reconstruction itself.

Reviewer 2 (Major Point 4): Lines 271-272, the authors wrote "our results demonstrate that training on data from multiple experimental conditions make it possible to reduce parameter uncertainty", I must say that it seems obvious: if you train on more data you reduce the uncertainty. What exactly is noticeable in the results? Is it that it actually works? Or how better it works, comapred to previous methods? Please clarify.

Response: We thank the reviewer for highlighting an area where our writing lacked precision. The point we intended to make is that our methodological contributions, which enable training on data from multiple conditions, had a tangible impact on the model. Specifically, they not only reduced uncertainty but also rendered several previously non-identifiable parameters identifiable and that this led to biologically more plausible predictions. We have rephrased the respective passage in the text.

Original text (Introduction): Finally, we demonstrate that the trained model accurately replicates oscillatory and arrested cell cycle dynamics in training data and predicts cell arrest states for cells at different starting points of the cell cycle as well as G1 arrest after growth factor depletion.

Revised text (Introduction): We demonstrate that only the trained model on both conditions accurately replicates oscillatory and arrested cell cycle dynamics in training data and predicts cell arrest states for cells at different starting points of the cell cycle as well as G1 arrest after growth factor depletion.

Original text (Results): Thus, our results demonstrate that training on data from multiple experimental conditions make it possible to reduce parameter uncertainty and build a biologically plausible model that performs correctly on a least a subset of conditions outside the training set.

Revised text (Results): Thus, our results demonstrate that training on data from multiple experimental conditions, particularly when these conditions provide informative variable dynamics, make it possible to significatively reduce parameter uncertainty and build a biologically plausible model that performs correctly on at least a subset of conditions outside the training set.

Reviewer 2 (Major Point 5): In the discussion, the authors argue (lines 304-306) that they "decided that a thorough exploration of the relationship between the particular pseudo-time embedding and the resulting mathematical model is beyond the scope of this study", so they do not investigate further the impact of using the pseudo-time technique in the resulting training of the models. I do believe, on the contrary, that this is in the scope of the manuscript. One cannot claim that a method is better than previous ones without investigating its limitations.

Finally, it is overall unclear what are the contributions of the authors: it seems it is a training accounting for multiple (here 2) conditions as well as a modificaton of the cell cycle model. Yet this is unclear, and I suggest it is clarified before a final decision is taken.

Response: Although this article introduces some modifications to the CMD method for obtaining pseudo-time trajectories, the primary aim is not to develop a new pseudo-time inference method. Rather, the main contribution of this work is to provide a framework that integrates pseudo-time ordering of multiplexed immunofluorescence data with mathematical modeling across both perturbed and unperturbed conditions, which, to our knowledge, has not been previously proposed. The model itself is also new: although it is based on a previously published structure, it incorporates substantial modifications that enable it to fit the multiplexed data, including both cell growth and arrest states. Overall, the study demonstrates how pseudo-time inference, with minor adaptations, can be combined with a trainable and identifiable model to explain dynamics of main cell cycle markers. This integrated approach successfully predicts cell-cycle arrest in G1 under low growth factor conditions, which were not included in the training data. We understand the confusion with the previous version of the manuscript and have revised it accordingly, with a corrected explanation now included in lines 101 – 113.

Original text (Abstract): We devise training strategies that are applicable to datasets where cells exhibit oscillatory as well as arrested dynamics and use them to train a cell cycle model on a dataset of MCF-10A mammary epithelial exposed to cell-cycle arresting small molecules.

Revised text (Abstract): We devise training strategies for mathematical models that are applicable to datasets where cells exhibit oscillatory as well as arrested dynamics and use them to train a cell cycle model on a dataset of MCF-10A mammary epithelial exposed to cell-cycle arresting small molecules.

Original text (Introduction): In this work, we extend a cc-CMD pseudo-time ordering [9] to enable model training using multi-condition data, thereby enabling pseudo-time ordering of perturbational data. Additionally, we develop novel model training techniques that can accommodate both oscillatory and steady-state constraints. We apply this framework to train a mathematical model of the cell cycle using previously described single-cell multiplexed immunofluorescence data [9]. We apply this framework to train a mathematical model of the cell cycle using previously described single-cell multiplexed immunofluorescence data on cell cycle progression and drug-induced cell arrest by palbociclib and nocodazole in MCF-10A cells [9]. Finally, we demonstrate that the trained model accurately replicates oscillatory and arrested cell cycle dynamics in training data and predicts cell arrest states for cells at different starting points of the cell cycle as well as G1 arrest after growth factor depletion.

Revised text (Introduction): In this work, we extend a cc-CMD pseudo-time ordering approach [9] to multi-condition data, thereby enabling pseudo-time ordering of perturbational data. Additionally, we develop a novel training technique for mathematical models that can incorporate oscillatory and steady-state constraints. We apply the extended cc-CMD approach along with this training method for mathematical models to multiplexed single-cell immunofluorescence data from MCF-10A cells [9], enabling the training of a mechanistic model of the cell cycle under both untreated and drug-induced arrested conditions by palbociclib and nocodazole. We demonstrate that only the trained model on both conditions accurately replicates oscillatory and arrested cell cycle dynamics in training data and predicts cell arrest states for cells at different starting points of the cell cycle as well as G1 arrest after growth factor depletion.

Original text (Results): Data from both untreated and treated conditions were used for parameter estimation. To encourage the model to exhibit stable oscillations under treated conditions, we concatenated four copies of the dynamic trajectories of all markers. To encourage model simulation to converge to a steady state under treated conditions, we concatenated 10 temporally equally spaced copies of the moving average marker concentrations at the cell cycle time of the corresponding arrest between the end of the first and the fourth cell cycle.

Revised text (Results): Data from both untreated and treated conditions were used for parameter estimation. The proposed model training approach builds on maximum likelihood estimation, with specific conditions for fitting oscillatory systems under drug-induced arrest. Specifically, to encourage the model to exhibit stable oscillations under treated conditions, we concatenated four copies of the dynamic trajectories of all markers. To encourage model simulation to converge to a steady state under treated conditions, we concatenated 10 temporally equally spaced copies of the moving average marker concentrations at the cell cycle time of the corresponding arrest between the end of the first and the fourth cell cycle.
